# Possible Sarcopenia and Impact of Dual-Task Exercise on Gait Speed, Handgrip Strength, Falls, and Perceived Health

**DOI:** 10.3389/fmed.2021.660463

**Published:** 2021-04-16

**Authors:** Reshma Aziz Merchant, Yiong Huak Chan, Richard Jor Yeong Hui, Jia Yi Lim, Sing Cheer Kwek, Santhosh K. Seetharaman, Lydia Shu Yi Au, John E. Morley

**Affiliations:** ^1^Division of Geriatric Medicine, Department of Medicine, National University Hospital, National University Health System, Singapore, Singapore; ^2^Department of Medicine, Yong Loo Lin School of Medicine, National University of Singapore, Singapore, Singapore; ^3^Biostatistics Unit, Yong Loo Lin School of Medicine, National University of Singapore, Singapore, Singapore; ^4^National University Polyclinics, National University Hospital System, Singapore, Singapore; ^5^Healthy Ageing Programme, Alexandra Hospital, National University Health System, Singapore, Singapore; ^6^Department of Geriatrics Medicine, Ng Teng Fong General Hospital, Singapore, Singapore; ^7^Division of Geriatric Medicine, Saint Louis University School of Medicine, St. Louis, MO, United States

**Keywords:** sarcopenia, grip strength, gait speed, dual-task exercise, frailty, perceived health, social isolation

## Abstract

**Background:** Sarcopenia is defined as a progressive age-related loss in muscle mass and strength affecting physical performance. It is associated with many negative outcomes including falls, disability, cognitive decline, and mortality. Protein enriched diet and resistance training have shown to improve muscle strength and function but there is limited evidence on impact of dual-task exercise in possible sarcopenia.

**Objective:** To evaluate impact of community-based dual-task exercise on muscle strength and physical function in possible sarcopenia defined by either slow gait (SG) or poor handgrip strength (HGS). The secondary aims include effect on cognition, frailty, falls, social isolation, and perceived health.

**Methods:** Community-dwelling older adults ≥60 years old were recruited from screening program intended to identify seniors at risk, and invited to participate in dual-task exercise program called HAPPY (Healthy Aging Promotion Program for You). One hundred and eleven participants with possible sarcopenia completed 3 months follow-up. Questionnaire was administered on demographics, frailty, sarcopenia, falls, perceived health, social network, functional, and cognitive status. Physical performance included assessment of HGS, gait speed, and Short Physical Performance Battery test (SPPB).

**Results:** The mean age of the Exercise group was 75.9 years old and 73.0% were women. The Exercise group had more female (73.0 vs. 47.5%), were older (75.9 vs. 72.5 years old), had higher prevalence of falls (32.4 vs. 15.0%), lower BMI (23.7 vs. 25.8), and education (4.0 vs. 7.2 years). The gait speed of the Exercise group increased significantly with significant reduction in the prevalence of SG and poor HGS. All components of SPPB as well as the total score increased significantly while the prevalence of pre-frailty and falls dropped by half. The risk of social isolation reduced by 25% with significant improvement in perceived health and cognition in the Exercise group. Significant impact on improvement gait speed and SPPB persisted after adjustment for baseline factors.

**Conclusion:** Dual-task exercise program is effective in improving gait speed, SPPB score, and reducing the prevalence of poor HGS with significant improvement in perceived health, cognition, and reduction in falls and frailty. Future prospective randomized control trials are needed to evaluate the effectiveness of dual-task interventions in reversing sarcopenia.

## Introduction

The world's older population ≥ 65 years old is projected to increase from 703 million in 2019 to 1.5 billion in 2050 causing an exponential increase in people with sarcopenia, frailty, cognitive impairment, and associated disability (1). Similarly, older persons ≥ 80 years old is projected to triple between 2019 and 2050 to 426 million (1). Sarcopenia is defined as a progressive age-related loss in muscle mass and strength affecting physical performance (2). It is classified as a disease under the World Health Organization (WHO)'s International Statistical Classification of Diseases and Related Health Problems (ICD) (3). The prevalence of sarcopenia ranges between 9 and 51%, and probable or possible sarcopenia between 26.3 and 73.3% depending on the case finding approach, population subgroup, and definitions used (4–8). Aging is a known risk factor for sarcopenia and the number of individuals with sarcopenia is projected to increase by 72.4% in Europe between 2016 and 2045 (9, 10).

Sarcopenia is associated with many negative health outcomes such as falls, fractures, functional decline, fear of falling, cognitive decline, depression, and mortality (2, 11). It is the precursor for physical frailty (9). While sarcopenia is a target for drug development, most drug therapeutic trials have been unsuccessful (12). The European Working Group on Sarcopenia in Older People recently updated the clinical definition and consensus diagnostic criteria for sarcopenia in 2018 incorporating low muscle mass, strength, and low physical performance (13). In recent years, there has been increasing emphasis on muscle quality where low muscle strength and poor performance rather than muscle mass are considered as principal determinants of adverse outcomes. The Sarcopenia Definition and Outcomes Consortium proposed for weakness defined by low handgrip strength (HGS) and slowness defined by low gait speed to be included in the definition of sarcopenia as both individually or in combination are associated with poor health outcomes (14).

Sarcopenia is often overlooked and undertreated in a busy clinical practice where a practical and effective screening tool like SARC-F can be used (15, 16). Slow gait (SG), prolonged chair-stand test, and/or poor HGS are included in many guidelines to diagnose possible sarcopenia to enable earlier case finding, assessment, and implement interventions to delay the decline or reverse the condition (13, 17).

Various studies have shown that muscle strength and function can be improved with protein enriched diet and resistance training exercise with variable impact on muscle mass (18). Gait and cognition share a common neural pathway, and dual-task exercise of varying intensity has shown to improve cognition and gait speed (19, 20). There is limited evidence on impact of dual task exercise on muscle strength and muscle function, and the type, intensity, and frequency of exercise in older adults with differing functional status is an emerging area of research (21). The aim of our study is to evaluate impact of community-based dual-task exercise on muscle strength and physical function in participants with possible sarcopenia. The secondary aims include effect on cognition, frailty, falls, social isolation, and overall perceived health.

## Methods

Community-dwelling older adults ≥ 60 years old in Singapore were recruited from population screening program intended to identify seniors at risk, e.g., pre-frail, frail, and those with cognitive impairment between August 2017 and December 2018. The publicity was through network of grassroots volunteers, senior activity centers, and words of peers. Phase 1 of the screening program was for general population, and those screened to be high risk were invited to participate in phase 2 screening and dual-task exercise program called HAPPY (Healthy Aging Promotion Program for You) conducted once or twice weekly within the neighborhood setting. The HAPPY program is adapted from “Cognicise,” a multi-component program designed by the National Center for Geriatrics and Gerontology (NCGG) in Nagoya, Japan (20). There are more than 80 different dual-task exercises of increasing complexity and intensity. The 60 min exercise sessions led by trained health coaches comprise of 20 min of stretching, warming up, and cooling down with 40 min of personalized dual-task training incorporating resistance, balance, aerobic, and cognitive tasks (e.g., marching, clapping, with step-up/down movement on the step-board with simultaneous naming/recalling tasks, subtracting, adding, and remembering the steps on the numbered colorful ladder). The implementation and types of exercises are described in a recently published paper (20).

**Figure 1:**
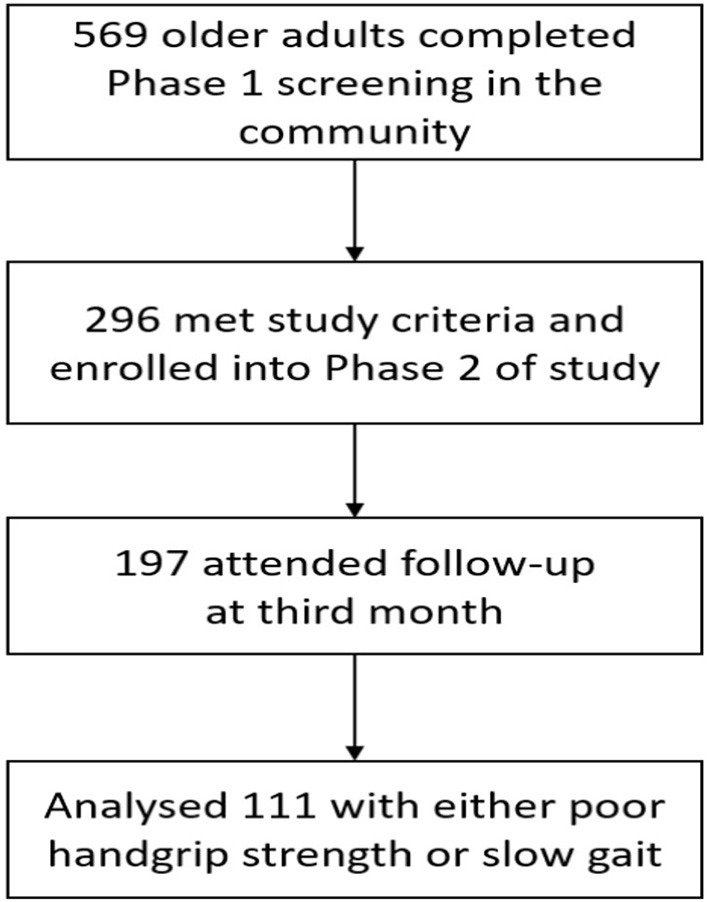
Flow of participant recruitment.

Exclusion criteria were diagnosis of dementia (Chinese Mini-Mental State Examination <18 or known diagnosis of dementia), wheelchair or bedbound, and living in a nursing home. A total of 569 seniors attended phase 1 screening, 296 participants were enrolled in phase 2 and complete follow up data was available for 197 participants at 3 months where 111 participants had either poor HGS or SG (Figure 1).

An interview questionnaire was administered by trained staff and included questions on demographics, frailty (FRAIL - Fatigue, Resistance, Ambulation, Illness, and Loss of Weight) (22), sarcopenia (SARC-F - lifting and carrying 10 pounds, walking across a room, transferring from bed/chair, climbing a flight of 10 stairs, and frequency of falls in the past 1 year) (16), falls, perceived health (EuroQol vertical visual analog scale) (23), social network (6-item Lubben Social Network Scale) (24), activities of daily living (ADL), and instrumental activities of daily living (IADL) using the KATZ ADL scale, and Lawton IADL scale, respectively (25, 26). Cognitive status was assessed using the modified Chinese Mini-Mental State Examination (cMMSE) which has been validated in the multi-ethnic groups locally and the Montreal Cognitive Assessment (MoCA) (27–29). The FRAIL questionnaire has been validated in Asian countries including locally, easy to administer and comparable with multidimensional deficit accumulation frailty index in predicting disability and mortality (22–31). The scores range from 0 to 5, where scores of 1–2 represent prefrail and 3–5 frail. Multi-morbidity was defined as presence of 2 or more of the following comorbidities: hypertension, hyperlipidemia, diabetes mellitus, heart disease, cancer, stroke, and lung disease.

Physical performance test comprised assessment of HGS, gait speed, and Short Physical Performance Battery test (SPPB). HGS was measured on the dominant arm using Jamar hand dynamometer in the seated position with elbow flexed at 90°. Maximum HGS was taken from two trials. Cut-offs of 28 kg for males and 18 kg for females were used to define poor HGS according to the Asian Working Group for Sarcopenia criteria (17). The SPPB was scored out of a total of 12 points and included components on balance, gait speed over 4 m, and five continuous chair-stand with a maximum of 4 points awarded for each component.

The controls aged ≥ 65 years old were recruited from a primary care practice in Singapore between October 2019 and December 2020. This group of participants did not participate in any intervention except being treated for their chronic diseases by their primary care physician and led their usual lifestyle.

Possible sarcopenia was defined as either having poor HGS or SG (<1 m/s) according to the Asian Working Group for Sarcopenia recommendations (17). Both the Exercise and Control groups had baseline assessments at 0 and 3 months. Ethics approval was obtained from Domain-Specific Review Board of National Healthcare Group, Singapore. All participants provided written informed consent.

### Statistical Analysis

Descriptive statistics were presented as mean values (standard deviation) for continuous variables and frequencies (percentages) for categorical variables. Differences in baseline characteristics between the Exercise and Control group were analyzed using Independent *T*-test on continuous variables and Chi-square on categorical variables. Change in outcome variables were calculated as difference between baseline and 3rd month time-point with positive values indicating improvement and negative values indicates decline. Paired sample *t*-test and McNemar were performed to determine statistical difference between baseline and 3rd month for continuous and categorical variables, respectively. To find out if changes were significantly different between the Exercise and Control group, linear regression was performed with change in individual outcomes variables as dependent variable and grouping (Exercise/Control), age, education level, and number of exercise sessions attended as independent variables in Model 1 and further, adjusted for corresponding baseline values, level of physical activity and presence of multi-morbidities in Model 2. Significance level was set at *p* < 0.05 and all analyses were analyzed using SPSS Version 26.0.

## Results

A total of 111 participants with either poor HGS or SG, participated in the HAPPY Program and completed assessment at 3rd month (Figure 1). The mean age of the Exercise group was 75.9 years old and 73.0% were women. The participants attended an average of 13 sessions over 3 months. Data of 40 participants recruited from the primary care practices with either HGS or SG, and did not participate in any intervention was used as “Control”. Comparison of baseline characteristics between the Exercise and Control groups are shown in Table 1. The Exercise group had more female (73.0 vs. 47.5%), were older (75.9 vs. 72.5years old), had higher prevalence of fall (32.4 vs. 15.0%), lower BMI (23.7 vs. 25.8), and education (4.0 vs. 7.2 years) than the Control group. Prevalence of chronic conditions was however higher in the Control group where almost 3 in 4 had multi-morbidities, 84.6% had hyperlipidemia, and 46.2% had diabetes mellitus. Majority of the participants in the Control group were pre-frail (97.4%), had higher cognitive score, better balance (3.7 vs. 3.1), and greater HGS (22.2 vs. 19.6kg).

**Table 1:** Baseline characteristics of participants in exercise and control groups.

	**Exercise** **(*n* = 111)**	**Control** **(*n* = 40)**	***P*-value**
Gender			**<0.01**
Male	30 (27.0)	21 (52.5)	
Female	81 (73.0)	19 (47.5)	
Age, years	75.9 ± 7.3	72.5 ± 5.5	**<0.01**
Ethnicity			**<0.01**
Chinese	105 (62.1)	30 (71.4)	
Malay	3 (42.9)	6 (85.7)	
Indian	3 (75.0)	4 (80.0)	
BMI, kg/m^2^	23.7 ± 4.2	25.8 ± 4.3	**<0.01**
Education, years	4.0 ± 3.7	7.2 ± 3.8	**<0.01**
Exercise sessions attended, number	13 ± 6	-	
At least 1 fall in past year	36 (32.4)	6 (15.0)	**0.035**
Fear of fall	83 (74.8)	31 (86.1)	0.073
Multi-morbidities, (At least two chronic conditions)	54 (48.6)	29 (74.4)	**<0.01**
Hypertension	65 (58.6)	27 (69.2)	0.239
Hyperlipidemia	57 (51.4)	33 (84.6)	**<0.01**
Diabetes mellitus	28 (25.2)	18 (46.2)	**0.015**
Heart disease	15 (13.5)	7 (17.9)	0.501
cMMSE score	25.0 ± 3.5	26.5 ± 2.8	**0.019**
Frail status			**<0.01**
Robust	31 (27.9)	0 (0.0)	
Prefrail	72 (64.9)	38 (97.4)	
Frail	8 (7.2)	1 (2.6)	
Sarcopenic	10 (9.0)	5 (14.3)	0.130
Pain (At least moderate)	57 (51.4)	17 (42.5)	0.337
Anxiety (At least moderately anxious/depressed)	7 (6.4)	5 (13.9)	0.159
EQ-5D VAS score	70.5 ± 15.1	70.2 ± 14.0	0.893
SPPB Total score	8.9 ± 2.4	9.6 ± 1.7	0.134
SPPB Balance score	3.1 ± 1.1	3.7 ± 0.6	**<0.01**
SPPB Gait score	3.4 ± 0.9	3.5 ± 0.6	0.334
SPPB Chair-stand score	2.5 ± 1.2	2.3 ± 1.2	0.391
Maximum grip strength, kg	19.6 ± 5.2	22.2 ± 5.7	**<0.01**
Poor handgrip strength	61 (62.9)	24 (61.5)	0.866
Maximum gait speed (4m), m/s	0.90 ± 0.28	0.87 ± 0.15	0.458
Slow gait	82 (75.2)	31 (86.1)	0.203
Takes part in moderate/vigorous intensity exercise weekly	30 (27.5)	24 (60.0)	**<0.01**
At risk of social isolation	59 (54.1)	14 (41.7)	0.195
At least one ADL impairment	10 (9.0)	6 (16.7)	0.200
At least one IADL impairment	27 (24.3)	9 (25.0)	0.935

*BMI, Body mass index; cMMSE, Chinese Mini-mental state examination; EQ-5DVAS, EuroQol Visual analog scale; SPPB, Short physical performance battery; ADL, Activities of daily life; IADL, Instrumental activities of daily life*.

*Data are presented as n (%), otherwise as mean (standard deviation). Bold values indicates statistically significant*.

**Figure 2:**
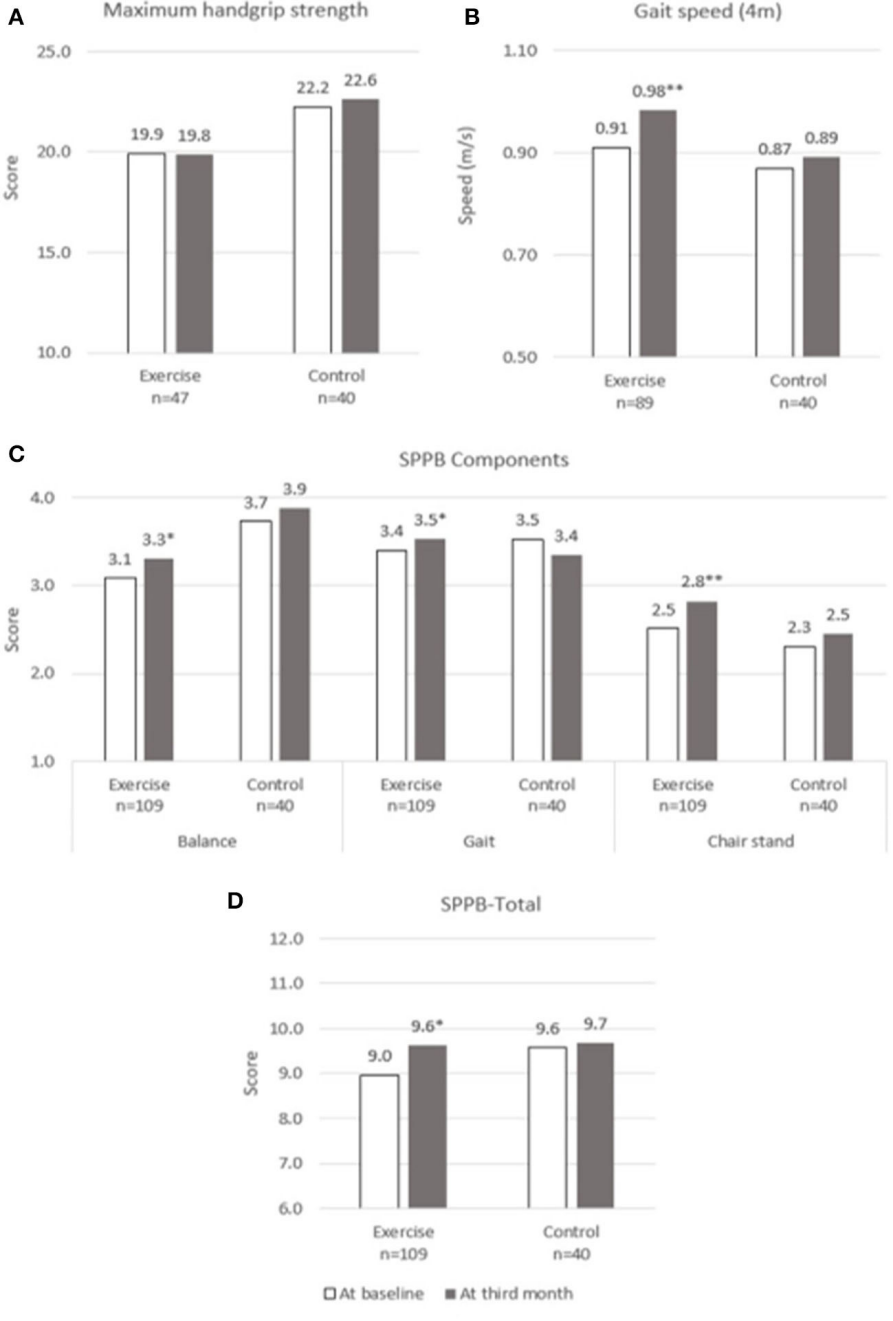
Physical performance at baseline and third month. **(A)** Maximum Handgrip Strength, **(B)** Gait speed, **(C)** SPPB Components, **(D)** SPPB-Total. SPPB, short physical performance battery. * and **indicate significant difference at *p* < 0.05 and *p* < 0.01, respectively between baseline and third month using paired sample *t*-test.

**Figure 3:**
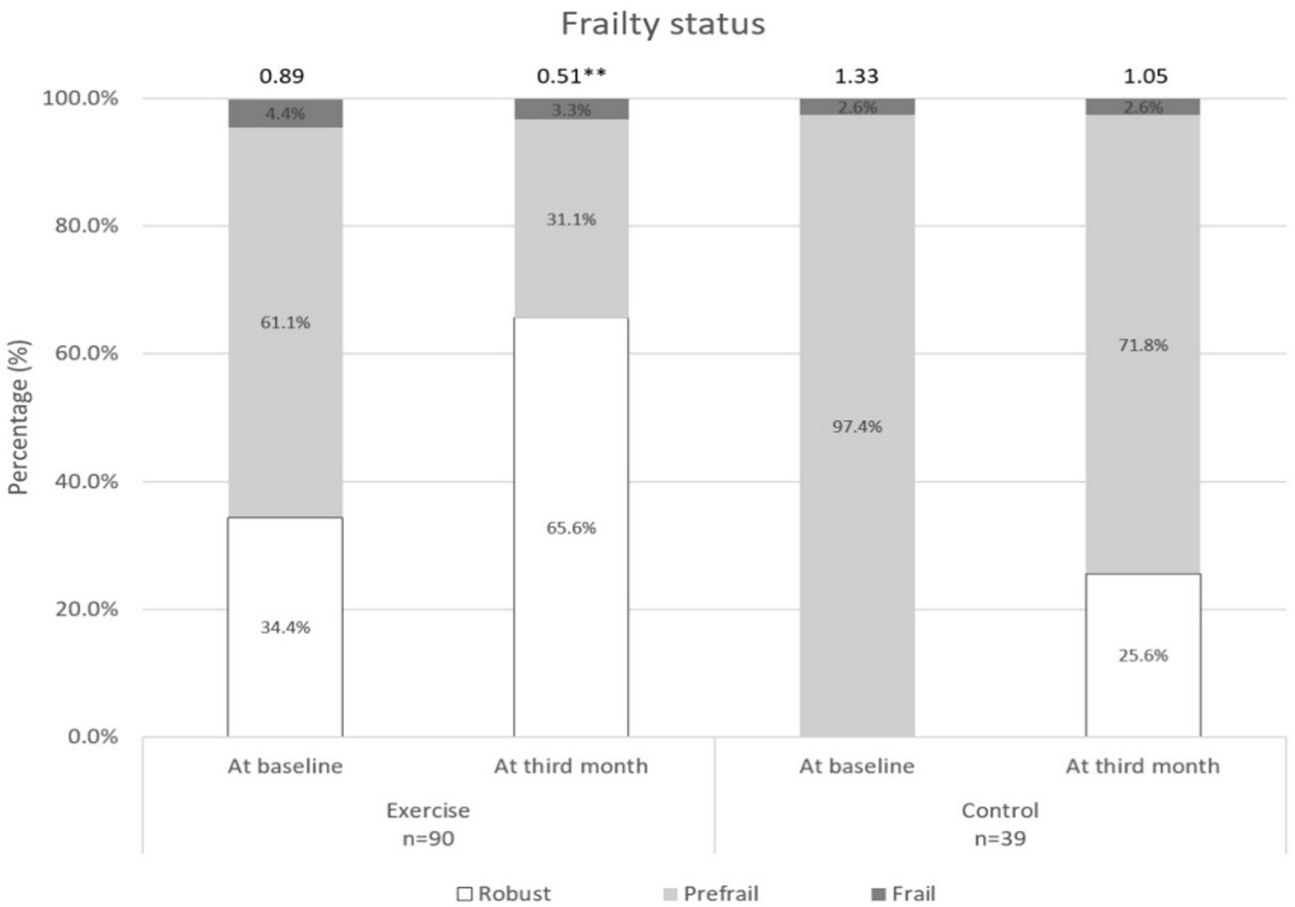
Frailty status at baseline and third month. **indicates significant difference at *p* < 0.01 between baseline and third month.

**Figure 4:**
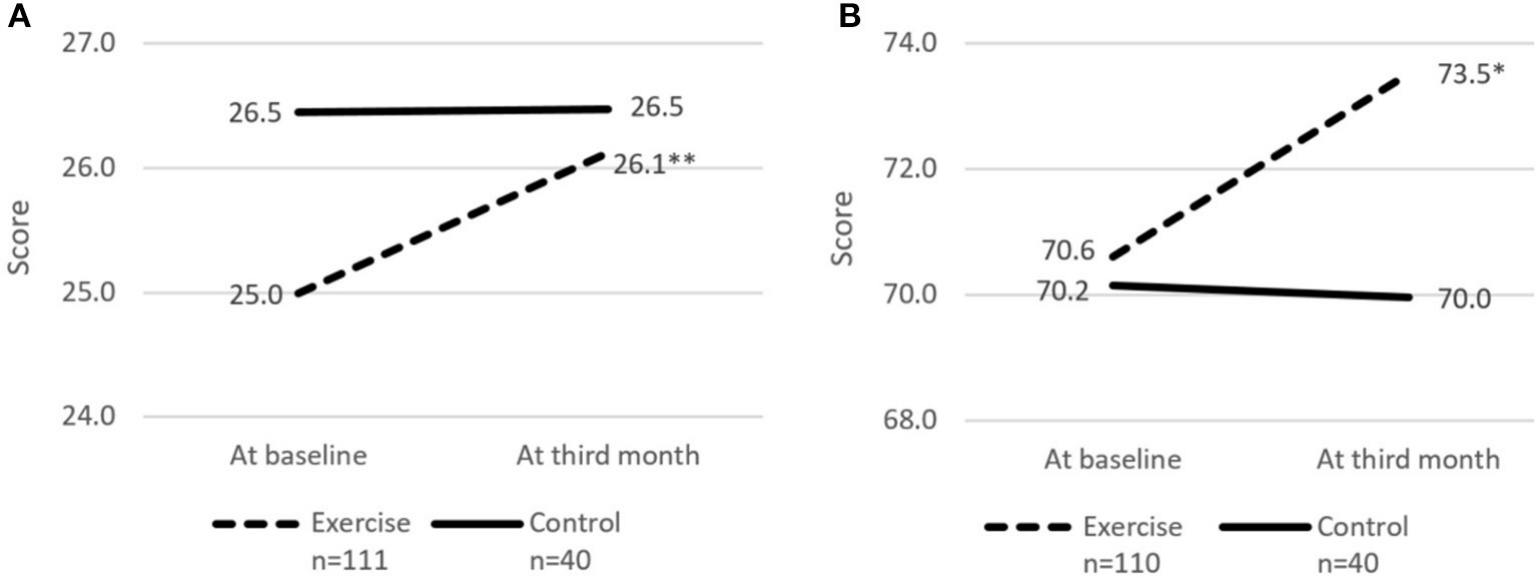
Cognitive score and perceived health rating at baseline and third month. **(A)** cMMSE. **(B)** Perceived health rating. cMMSE, Chinese Mini-mental state examination. Perceived health rating derived from EuroQol - Visual Analog Scale. * and **indicates significant difference at *p* < 0.05 and *p* < 0.01, respectively between baseline and third month using paired sample *t*-test.

**Figure 5:**
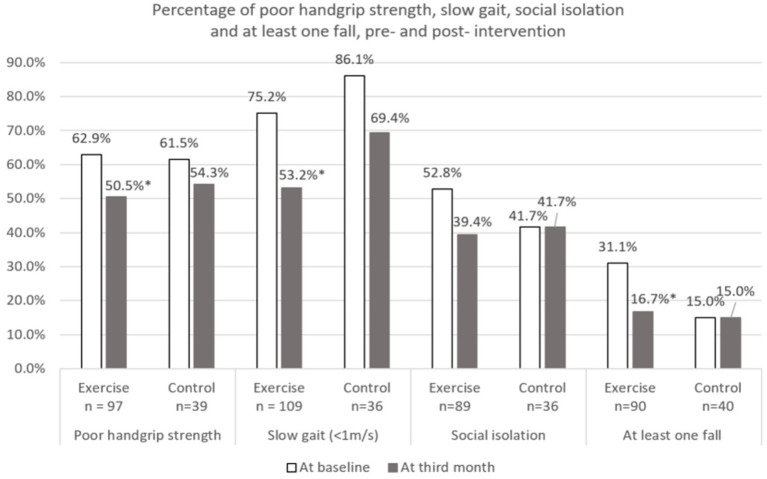
Percentage of poor physical performance, social isolation, and at least one fall at baseline and third month. *Indicates significant difference at *p* < 0.05 between baseline and third month using McNemar test. Poor handgrip strength defined using Asian Working Group for Sarcopenia (2019) (Cut-off: Male < 28 kg and Female < 18 kg). Social isolation defined using 6-item Lubben social network scale (Cut-off: < Score of 12).

Changes over the 3 months for both groups are shown in Figures 2–5. Several significant improvements were observed in the Exercise group. The maximum gait speed of the Exercise group increased significantly after 3 months from 0.91 (95% CI 0.86–0.96) to 0.98 (95% CI 0.92–1.04) m/s, and the prevalence of SG decreased from 75.2% (95% CI 66.2–82.4%) to 53.2% (95% CI 44.3–62.9%). There was no significant change in maximum HGS for both the groups but the prevalence of poor HGS decreased significantly in the Exercise group. All components of SPPB as well as the total score increased significantly in the Exercise group while the prevalence of pre-frailty dropped by half from 61.1% (95% CI 50.9–70.5%) to 31.1% and falls 31.1% (95% CI 22.3–41.2%) to 16.7% (95% CI 10.1–25.4%).

The risk of social isolation reduced from 52.8% (95% CI 42.5–63.0%) to 39.4% (95% CI 29.7–49.7%) in the Exercise group. There was significant within group improvement of self-perceived health rating from 70.6 (95% CI 67.7–73.5) to 73.5 (95% CI 70.6–76.5) for the Exercise group while rating for the Control group remained unchanged. Significant increase in cognitive score (cMMSE) was seen in the Exercise group from 25.0 (95% CI 24.3–25.6) to 26.1 (95% CI 25.4–26.8) while the score remained the same for the control group after 3 months.

When comparing differences between the Exercise and Control groups, the Exercise group showed significantly greater improvement in cMMSE (Unstandardized β = 1.11, 95% CI = 0.13–2.10, *p* = 0.027) before adjustment (Table 2). Model 1 was adjusted for age, education level, and number of sessions attended and model 2 was further adjusted for corresponding baseline scores/measurements, physical activity level and presence of multi-morbidities. Improvement in maximum gait speed and total SPPB score were significantly greater in the Exercise group both in Model 1 (Unstandardized β = 0.15, 95% CI = 0.03–0.26, *p* = 0.017 and Unstandardized β = 1.31, 95% CI = 0.35–2.26, *P* < 0.01) and Model 2 (Unstandardized β = 0.17, 95% CI = 0.05–0.30, *p* < 0.01 and Unstandardized β = 1.13, 95% CI = 0.23–2.03, *P* = 0.014).

**Table 2:** Intervention effect on change in cognition, physical performance, and overall health rating.

**Factors**	**Unstandardized β-coefficient**		
	**Unadjusted**	**Model 1**	**Model 2**
Maximum handgrip strength	−0.24 (−1.52–1.04) 0.709	0.16 (−0.14–0.09) 0.643	−0.79 (−2.93–1.35) 0.466
Maximum gait speed (4 m)	0.05 (−0.03–0.13) 0.189	**0.15 (0.03–0.26) 0.017**	**0.17 (0.05–0.30) <0.01**
EQ-5D VAS	3.12 (−2.40–8.64) 0.266	−0.53 (−0.92–8.35) 0.906	3.84 (−4.41–12.10) 0.359
cMMSE	**1.11 (0.13–2.10) 0.027**	0.57 (−1.01–2.15) 0.475	0.55 (−1.03–2.13) 0.492
SPPB total	0.56 (−0.06–1.16) 0.074	**1.31 (0.35–2.26) <0.01**	**1.13 (0.23–2.03) 0.014**
Frailty score	0.02 (−0.33–0.37) 0.896	−0.24 (−0.81–0.34) 0.416	0.09 (−0.39–0.56) 0.720
Lubben social network score	0.94 (−0.77–2.65) 0.280	2.65 (−0.13–5.42) 0.061	2.19 (−0.53–4.92) 0.114

*Unstandardized β-coefficients (95% CIs) and p-values are shown from linear regression models with change in scores/readings as dependent variable. Model 1 is adjusted for age, education level, and number of sessions attended. Model 2 is further adjusted for corresponding baseline scores/measurements, physical activity level, and presence of multi-morbidities. Bold font indicates p-values <0.05*.

## Discussion

With population aging, maintaining functional and cognitive ability, improving quality of life, and reducing social isolation should be every country's priority. The Decade of Healthy Aging Report has highlighted on the need to design national programs on age-friendly cities and community to add life to years (32). Many countries are focusing on multi-strategic cost-effective population programs to maintain functional and cognitive ability (21, 33, 34). Older adults are heterogenous and may not be able to participate in high intensity resistance exercise. Determining the threshold and optimal levels of physical activity that are necessary for healthy aging or disease management is crucial for older persons with declining intrinsic capacity. The Healthy Aging Promotion Program for You (HAPPY) is a community-based tailored dual-task exercise program for older adults led by health coaches (HC) to promote healthy aging has shown to improve function and cognition in at risk older adults (20). Three months of dual-task exercise program for older adults with possible sarcopenia defined by poor HGS or SG showed significant improvement in gait speed, balance, chair-stand, frailty status, cognition, perceived health, and reduction in falls compared with control. The significant improvement in gait speed and total SPPB scores persisted even after adjusting for confounding factors such as age, education, multimorbidity, and physical activity.

Various guidelines have defined possible or probable sarcopenia as the presence of weakness or slowness as a reflection of muscle quality to enable upstream interventions before the onset of sarcopenia or severe sarcopenia (13, 14, 17). SARC-F which has a very high specificity, fast, and practical has been used as a case-finding tool in the community and hospital setting (35, 36). HGS is a good surrogate for muscle strength and gait speed for physical performance where both are well-established markers of biological aging and intrinsic capacity (37). Various studies have found that higher gait speed, HGS, and shorter time to complete chair-stand test are associated with independent aging (38). HGS is reproducible, reliable, and can be measured easily using inexpensive portable device. Low HGS is a known predictor of poor health outcomes such as falls, mobility limitation, functional impairment, and mortality in community dwelling older adults. The various guidelines on diagnosis of sarcopenia have different cut-point for poor HGS depending on gender, age, ethnicity, and population. Poor HGS can also be affected by occupation, depression, motivation, pain, and arthritis of the hands which were not evaluated in our study (17, 39).

Sarcopenia and cognition are closely related, both associated with aging and still an area of ongoing research. Sarcopenia is a risk factor for metabolic syndrome as skeletal muscle plays a crucial role in body's glucose metabolism, and both conditions either in isolation or together are associated with increased prevalence of cognitive impairment (40). Brain-derived neurotrophic factor released by contracting skeletal muscles is responsible for synapse and structural connectivity. The individual components of sarcopenia such as SG and muscle strength are known to be associated with cognitive impairment. In a study by Buchman et al. there was 9% increased risk of AD with each 1-lb annual decline in HGS (41). Gait speed is a well-recognized predictor of dementia especially in those with underlying cognitive impairment (42). Gait is a complex activity which involves planning and interplay between the central and peripheral nervous system, body systems e.g., cardiovascular, respiratory and musculoskeletal systems, fitness, and vision. Gait and cognition share similar neural pathway involving the corpus callosum, prefrontal, parietal, and temporal areas. SG is associated with impairment in many cognitive domains including attention, executive function, language, construction, abstraction, and orientation (43). More than one third male and one-half female ≥ 80 years old have SG (43). Strength training and aerobic exercises between 10 and 24 weeks have shown to improve cognition in prior studies (44). Dual-task exercise incorporating cognitive task and physical exercise showed increased activation in Broca's area, corresponding area on right hemisphere, widespread cortical activation across fronto-temporo-parietal areas and prefrontal cortex (45, 46). The participants in the dual-task exercise group in our study did improve in gait speed, cognition, balance, and chair-stand after 3 months. Previous studies have shown that dual-task exercises are better than single-task in improving gait speed, cadence, and other cognitive variables (47).

There was significant reduction in falls in the Exercise group at 3 months. Older adults are at higher risk of falls during dual task activities such as talking while walking. Motor control and walking requires intact neural system, attention, and planning. Cognitive impairment affects the planning and multisensory integration processes for gait in turn causing falls. Participation in cognitive activities is effective in improving neuromotor performance and possibly reducing falls with shorter foot reactive time and faster gait speed (48). The Exercise group also had significantly better perceived health at 3 months and reduction of social isolation by 25%.

Decline in physical performance during hospitalization and up to 3 months post discharge has been attributable to loss of muscle mass and muscle strength, and exercise programs in the hospital and post discharge is crucial to reduce the impact of acute illness on physical performance and enhance recovery (21, 49, 50). Most studies have focused on high intensity resistance training. Based on findings from our study and a recent study by Martínez-Velilla et al. (50), exercises tailored to individual's functional status can also be introduced in the hospital and post discharge to reduce the impact of post-hospitalization functional decline.

Covid-19 pandemic and associated measures such as social distancing and lockdown has resulted in reduction of physical activity and alteration in dietary habit increasing sarcopenia prevalence (51). While previous studies have emphasized on the need of resistance exercise and protein supplement, our study is the first to document improvement in gait speed, with reduction in the prevalence of SG and poor HGS after 3 months of dual-task exercise. While there are many studies evaluating web-based multi-domain interventions in the home setting, the inclusion of web-based dual-task exercises combined with resistance exercise, and protein enriched diet needs to studied to guide Public Health authorities on measures to prevent sarcopenia during future pandemics and lockdown (52).

There is a constant debate on how do we define outcomes which are meaningful for participants as statistically significant may not necessarily translate into being clinically meaningful and vice versa (53). Improvement in gait speed of 0.07 m/s and SPPB 0.5 points are considered clinically meaningful change based on ICFSR Task Force perspective and our study participants did show improvement of 0.07 m/s in gait speed and 0.6 points in SPPB which were also statistically significant. There was no significant change in HGS between the groups but greater significant reduction in the prevalence of poor HGS in the Exercise group. The possible explanation could be due to higher numbers of male in the Control group whom are known to have higher HGS and higher cut-off for poor HGS, and gender variation between the two groups. It is not known if 3 months is too short to notice any improvement in HGS as other multicomponent studies have shown similar findings (54).

The main strengths of our study are inclusion of community dwelling older adults with either poor HGS or SG, and the increasing complexity dual-task exercises conducted in neighborhood setting by trained health coaches suited to their functional performance. However, there are several limitations. The initial selection criteria to participate in the dual-task exercise were either prefrail, frail, or underlying cognitive impairment (cMMSE ≤ 26). SG and poor HGS is a subgroup analysis and were not the primary inclusion criteria. In addition, our interventions were not homogenous nor fully standardized, conducted twice weekly in slightly more than half of the participants, and the type and intensity of the dual-task exercises varied each week at health coaches discretion. Despite this, there were significant improvements and possibility of greater improvement if the exercises were conducted twice weekly in the Exercise group as shown in the entire group (20). Our study was not a randomized controlled trial but the recruitments were from different sites, and the final results were adjusted for differences between the 2 groups.

Despite the limitations, our study has generated a few interesting findings. Gait speed and cognition are closely associated, and 3 months dual-task exercise of varying intensity and complexity is effective in reducing the prevalence of SG, poor HGS, improving gait speed, frailty, perceived health, cognition, reducing falls, and social isolation. Population level screening with SARC-F with necessary targeted intervention may help reduce prevalence of sarcopenia and associated complications, reduce falls, and improve quality of life (33).

Future prospective randomized studies are needed to compare aerobic, high intensity strength training, and dual-task exercise with or without high protein diet in older people with differing functional status on the effect of muscle strength, performance, and muscle mass.

## Conclusion

Possible sarcopenia defined by either SG or poor HGS are known to be associated with poor outcomes. High intensity resistance exercise and high protein diet are known to improve muscle strength and performance. Dual-task exercise program of varying type and increasing intensity is useful in improving gait speed, SPPB scores, and reducing the prevalence of poor HGS with significantly improved perceived health. Future prospective randomized control trials are needed to evaluate the effectiveness of dual-task interventions in reversing sarcopenia and associated complications.

## Data Availability Statement

The raw data supporting the conclusions of this article will be made available by the authors, without undue reservation.

## Ethics Statement

The studies involving human participants were reviewed and approved by Domain Specific Review Board of National Healthcare Group. The patients/participants provided their written informed consent to participate in this study.

## Author Contributions

Conceptualization was performed by RM, JL, and JM. Funding acquisition and writing of original draft was performed by RM. Statistical analysis was performed by RM, YC, and JL. Methodology, project administration, review, and editing were performed by RM, JL, RH, SK, SS, LA, and JM. All authors contributed to the article and approved the final draft.

## Conflict of Interest

The authors declare that the research was conducted in the absence of any commercial or financial relationships that could be construed as a potential conflict of interest.
